# Ten-Year Change in Disorders of Consciousness: A Bibliometric Analysis

**DOI:** 10.3390/medicina59010078

**Published:** 2022-12-29

**Authors:** Xiaochun Zheng, Chengwei Xu, Shuiyan Li, Wanchun Wu, Qiuyi Xiao, Qiuyou Xie

**Affiliations:** Joint Research Centre for Disorders of Consciousness, Department of Rehabilitation Medicine, Zhujiang Hospital, Southern Medical University, Guangzhou 510515, China

**Keywords:** disorders of consciousness, bibliometric analysis, web of science, visualization, CiteSpace

## Abstract

*Objectives*: Disorders of consciousness (DoC) is a dynamic and challenging discipline, presenting intriguing challenges to clinicians and neurorehabilitation specialists for the lack of reliable assessment methods and interventions. Understanding DoC keeps pace with scientific research is urgent to need. We quantitively analyzed publications on DoC over the recent 10 years via bibliometrics analysis, to summarize the intellectual structure, current research hotspots, and future research trends in the field of DoC. *Methods*: Literature was obtained from the Science Citation Index Expanded of Web of Science Core Collection (WoSCC). To illustrate the knowledge structure of DoC, CiteSpace 5.8.R3 was used to conduct a co-occurrence analysis of countries, institutions, and keywords, and a co-citation analysis of references and journals. Also, Gephi 0.9.2 contributed to the author and co-cited author analysis. We found the most influential journals, authors, and countries and the most talked about keywords in the last decade of research. *Results*: A total of 1919 publications were collected. Over the past 10 years, the total number of annual publications has continued to increase, with the largest circulation in 2018. We found most DoC research and close cooperation originated from developed countries, e.g., the USA, Canada, and Italy. Academics from Belgium appear to have a strong presence in the field of DoC. The most influential journals were also mainly distributed in the USA and some European countries. *Conclusions*: This bibliometric study sheds light on the knowledge architecture of DoC research over the past decade, reflecting current hotspots and emerging trends, and providing new insights for clinicians and academics interested in DoC. The hot issues in DoC were diagnosing and differentiating the level of consciousness, and detecting covert awareness in early severe brain-injured patients. New trends focus on exploring the recovery mechanism of DoC and neuromodulation techniques.

## 1. Introduction

Consciousness refers to an individual’s cognitive abilities towards self and environment, including wakefulness and awareness [1]. The former means one can be receptive to external stimulation, while the latter means being able to respond to stimulation. Disorders of consciousness (DoC) is a common complication after severe brain injury [2,3], in which patients’ ability to awaken and perception of self and environment stimuli reduces or lose to varying degrees. DoC mainly includes the vegetative state (VS), also known as the unresponsive wakeful state (UWS), and the minimally conscious state (MCS). VS/UWS is an awakened state without clinical awareness, showing unconsciousness of oneself and the environment. In these patients, their eyes are open, accompanied by blinking; however, the circadian sleep-wake cycle is not observed [4]. In contrast, MCS patients show clear signs of non-reflex cortically mediated behavior in response to environmental stimuli, which occurs inconsistently but repetitively, and is a key criterion to distinguish MCS from VS [5]. With recent advances in critical care medicine, the number of patients who have survived the acute phase of injury and fallen into unconsciousness is increasing, and the management of such patients has been a major clinical and neuroscientific challenge [6]. The past decade has seen much DoC-related literature emerging but is not limited to various areas of neurobehavior, neuroimaging, and neurophysiology.

Bibliometrics, a new evidence-based research analytical framework that analyzes the publication patterns of books, articles, and other publications, particularly in scientific content, through statistical methods [7]. Visualization analysis enables researchers to quickly stay on top of the latest research hotspots and trends in their research fields, which represents important players in many research fields such as transportation, medicine, education, and neuroscience. 

To our knowledge, there has been only one DoC-related bibliometric analysis of neuroimaging trends analyzed in the literature published between 2002 and 2011 [8], and no bibliometrics analyses have been performed yet. Hence, by collecting data related to DoC research published after 2012, this study aims to provide an update on previous research and reveal the development of DoC as a whole, summarize the research status, and strive to get a clearer trend of DoC development, results from our analysis may better provide present new ideas for future research and clinical applications of DoC in neuroscience. Our research mainly answers the following three questions:

(1) What is the knowledge structure of the current research system on DoC?
(2) What is the focus of current research on DoC?
(3) What are the possible directions for future research on DoC?

## 2. Materials and Methods

### 2.1. Data Collection 

Related literature was collected by two authors (Xu and Zheng) through Web of Science (WoS) publication data on 6 July 2022, using the following search terms: topic = (“disorders of consciousness” or “vegetative state” or “unresponsive conscious state” or “minimally conscious state” or “Emergence from MCS”), index = Science Citation Index Expanded (SCI-EXPANDED). We included literature published from 2012 to the present. Papers were then exported as plain text files(.txt) and imported into CiteSpace to remove duplicated papers.

### 2.2. Inclusion Criteria 

**Figure 1:**
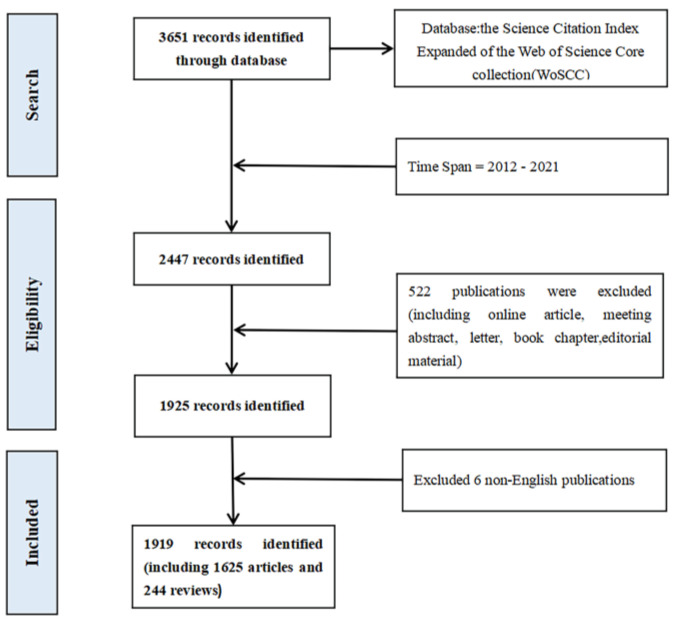
Flow chart of the retrieval strategy in this study.

The inclusion criteria are displayed in Figure 1. To eliminate bias, two authors were responsible for screening. Consistency of more than 90% was required. It is only considered if the title or abstract contains the word “disorders of consciousness” or “vegetative state”, or “unresponsive conscious state”, or “minimally conscious state”, or “Emergence from MCS”. Articles not related to DoC were excluded. This study only dealt with "article type" or "review" published in English. Finally, a total of 1919 published papers were included. For detailed information, please see Supplementary Materials, Spreadsheet S1.

### 2.3. Analytical Methods

An extensive survey of related studies was conducted to provide a comprehensive picture of DoC research. CiteSpace is the most widely used analytical software in bibliometric research [9]. CiteSpace 5.8.R3 was used for data analysis and visualization, it was used to construct country analysis and keywords co-occurrence map, and detect the citation burst of keywords and co-cited references. Additionally, we used the BibExcel software to extract the co-occurrence relationship (across authors), and then the relations were visualized by the Gephi 0.9.2 to form a collaboration network (https://gephi.org/, accessed on July 2022). We used linear regression to assess the trend of annual publications over time. Simply analyzing the publications published by authors or journals each year is a simple measure, to better describe the primary picture of the study, we added the 2021 impact factor (IF) and 2021 Journal Citation Reports (JCR) published in June 2022 to measure the influence of a journal. Meanwhile, we used H-index to describe the influence of an individual, country, or institution [10], and the number H represented the minimum number of citations.

## 3. Result

### 3.1. Publication Output and Time Trend

**Figure 2:**
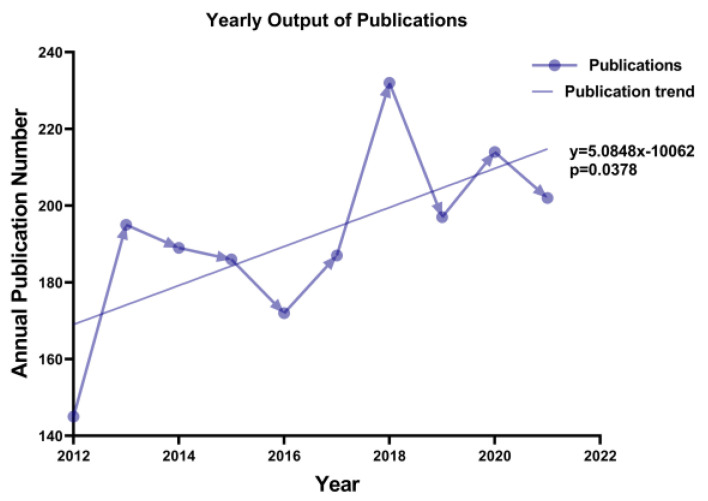
Annual publication outputs and time trend in DoC publications.

The overall distribution of annual publications and trends over time were shown in Figure 2. There were 1919 published DoC-related articles in the past decade. Annual publications of DoC-related articles showed a fluctuating trend, but the overall trend showed an upward trend, with 145 references in 2012 and 202 references in 2021, published the most in 2018, indicating a year of rapid development in the field of DoC. Growth trends were consistent with the linear forecast relationship, namely, y = 5.0848x − 10062 (*p* < 0.05), where y represents the annual publication number, and x represents the year.

### 3.2. Author Cooperation and Co-Authors Analysis

**Figure 3:**
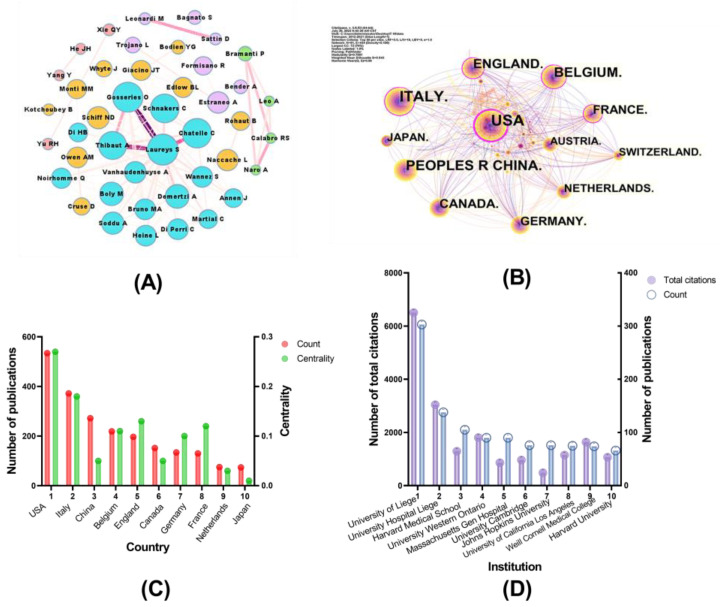
Analysis of country and institution and author distribution. (**A**) Co-occurrence analysis of author collaboration network. Node represents author. Line represents cooperation, closer the collaboration, thicker the line. Every color refers to a cooperation group, the area of the circle represents publications. (**B**) Analysis of co-occurrence of country distribution. Node represents country. Line represents cooperation. The purple outer circle represents higher centrality. The size of nodes refers to the number of publications. (**C**) The top 10 the most influential countries of DoC research and their centrality. (**D**) The top 10 most influential institutions of DoC research and their total citation.

A total of 2714 authors contributed to DoC-related studies. A network of the academic cooperation of authors was presented in Figure 3A. Laurys S was the most dominant contributor to the cooperation network and the most published author followed by Gosseries O, and then Owen AM in this field (Table 1). 

Close collaborations were found between Laureys S and Gosseries O, both from the University of Liege, Belgium. As for co-cited authors, Giacino JT was the most productive co-cited author, followed by Laurys S and Schnakers C. Laurys S, Schnakers C, and Owen MA were the authors with the highest H-index among both authors and co-authors, suggesting that the three authors are among the most influential researchers in the field of DoC.

### 3.3. Countries and Institutions Analysis

A total of 91 countries participated in the DoC study. Figure 3B showed the results of the country co-occurrence analysis, node represents country and line means cooperation. The network revealed the USA had the highest centrality and published the most, and close collaborations were found between USA and Belgium, Italy, and Canada. The top 10 most active countries were reported in Figure 3C. Among the top 10 countries, USA, Italy, UK, France, Belgium, and Germany represented countries with high centrality (>0.1), indicating that these countries had a significant influence in this field. 

Additionally, we identified 1885 institutions as a source for DoC research publications. University of Liege, University Hospital Liege, and Harvard Medical School work were the most productive institutions (Figure 3D) and inferred they were the most closely related to other institutions. 

### 3.4. Journal Distribution and Co-Citation Analysis

Journals and co-cited analysis focus on finding influential journals in the DoC field. Table 2 presented the top 10 journals in the DoC field and co-cited journals. In the past decade, Brain Injury had the highest number of publications (IF = 2.167), followed by Frontiers in Neurology (IF = 4.086) and Frontiers in Human Neuroscience (IF = 3.473). Neurology (1215 citations, IF = 11.8), New England Journal of Medicine (930 citations, IF = 176.079), and Archives of Physical Medicine and Rehabilitation (911 citations, IF = 4.06) were cited the most. New England Journal of Medicine had the highest Impact Factor (IF = 176.07).

### 3.5. Reference Co-Citation Analysis

Co-citations references are the foundation of a field of study. Reference co-citation analysis enables the exploration of the hot spots in the DoC field, making it convenient for researchers to discover research hotspots for the first time [10]. A total of 1919 original papers were cited 774 times. As shown in Table 3, the first rank co-cited reference in the top 10 was published in the New England Journal of Medicine and written by Monti MM et al. fMRI was used to observe the brain activation of patients with DoC in mental-imagery tasks to detect residual consciousness [11], suggesting that fMRI may be a useful tool to establish basic communication among patients who seem unresponsive in bedside. The second co-cited reference was published by Laureys S et al. [12]. Considering the term “vegetative state” vegetative state may have a negative connotation for many physicians and medical workers, they proposed a new name for patients who were in the vegetative state called “unresponsive wakefulness syndrome”. The third co-cited reference was published by Schnakers S et al. [6]. They used JFK Coma Recovery Scale-Revised (CRS-R) as an assessment tool, which significantly improved the classification accuracy of DoC patients compared with clinician experience and consensus, and recommended the use of standardized neurobehavioral assessment in clinical diagnosis, which can greatly reduce the rate of misdiagnosis.

The centrality can also detect the most critical article in the DoC field. Higher centrality means the article is more critical. The highest one was written by Kondziella D et al. [19], who systematically reviewed both active and passive paradigms, which used fMRI or electroencephalogram, to probe whether these two tools can distinguish between MCS and VS sensitively. It showed that active paradigms may underestimate patients’ actual level of consciousness, while the passive paradigms seemed to have greater specificity. Notably, MCS patients performed well in both paradigms.

**Figure 4:**
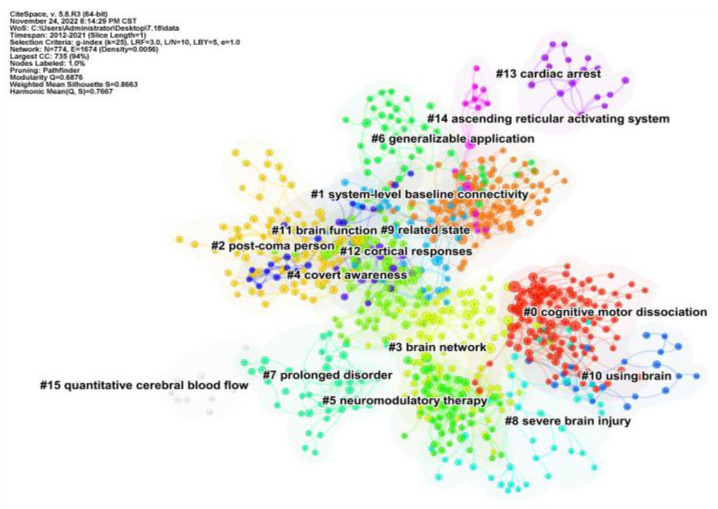
Co-cited references cluster analysis co-occurrence map. Node represents reference, every color represents one cluster. Label means cluster theme, the label number is sorted by the size of the cluster.

The co-cited references are clustered and analyzed (Figure 4), and the five largest clusters include cognitive motor dissociation, system-level baseline connectivity, post-coma person, brain network, and covert awareness. Each cluster represents a key topic of interest in the field of DoC. The network contains 774 nodes and 1674 links. The weighted mean silhouette score is 0.8663, which shows that clusters had a good consistency.

### 3.6. Keywords Co-Occurring Analysis and Citation Bursts

**Figure 5:**
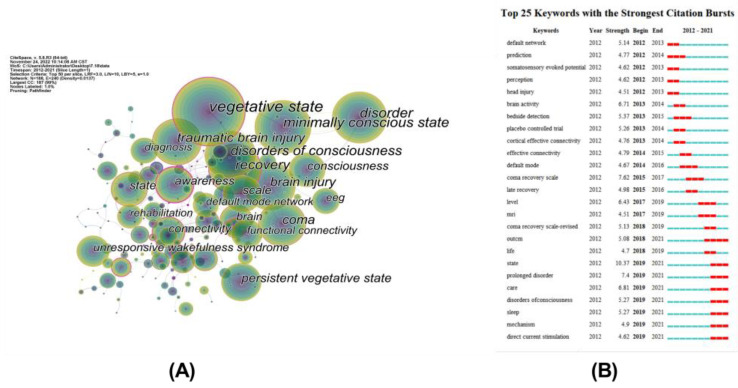
Analysis of keywords. (**A**) Keyword co-occurrence analysis. Node represents keywords. The purple outer circle represents higher centrality. The size of nodes refer to the frequency of keywords. (**B**) The top 25 keywords with the strongest citation bursts. Ten bars represent the years 2012–2021. The red segment of the blue line is the years of burst duration.

Keyword co-occurrence analysis is to find the hotspots of current research. High-frequency keywords could reflect research hotspots. Table 4 showed the top 10 keywords ranked by records and centrality and the visualization of the results are presented in Figure 5A. The top 10 keywords ranked by numbers were vegetative state, minimally conscious state, disorder, disorders of consciousness, disorder, disorders of consciousness, recovery, traumatic brain injury, brain injury, coma, persistent vegetative state, awareness. Additionally, among the top 10 keywords of centrality, awareness ranked first (centrality = 0.56), followed by connectivity (centrality = 0.38), own name (centrality = 0.37).

Keyword citation bursts can also be used as an indicator of research trends [20]. A total of 180 keywords were included in this analysis. Figure 5B showed the top 25 keywords with the strongest citation bursts. The blue line represented the time interval, and the red line represented the period in which the keyword appeared. From 2019 to 2021, the most recent burst keywords included state, prolonged disorder, care, disorders of consciousness, sleep, mechanism, and direct current stimulation. The keyword “state” is the highest one (10.37).

**Figure 6:**
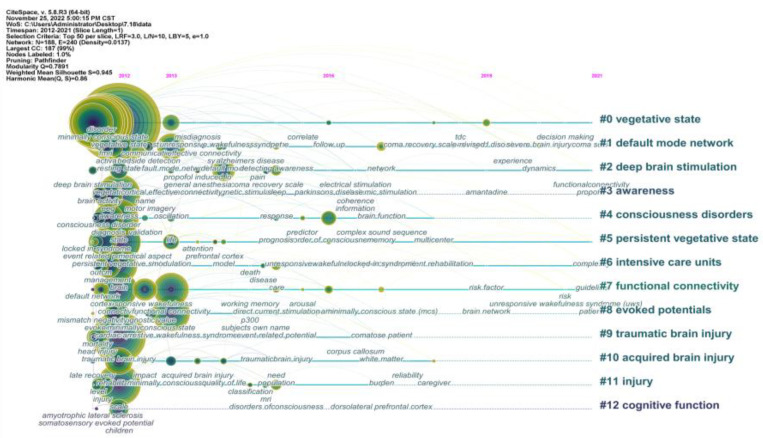
The timeline view of keywords. The nodes represent keywords, and the position on the horizontal line represents the time, the word under the line represents the keyword appearing in this period. The label on the right refers to the theme of the cluster.

To visualize the development trends of keyword clusters and delineate the relationship between clusters keywords, a timeline view of keywords was performed (Figure 6). Alternatively, 5 clusters are the most concerned research hotspots, including cluster #0 vegetative state, cluster #2 deep brain stimulation, cluster #5 persistent vegetative state, cluster #6 intensive care units, and cluster #7 functional connectivity.

## 4. Discussion

### 4.1. Intellectual Structure of Publications on DoC 

In this study, we quantitively analyzed the publications from the recent 10 years via Bibliometrics tools to explore the hotspots and trends in the DoC field, depict a knowledge map of disorders of consciousness research from author, country, institution, journal distribution, and keyword co-occurrence analysis. 

A total of 1919 publications were collected from WoS. Although the number of articles published fluctuates every year, the overall number is increasing and peaked in circulation in 2018. Laureys S was the most prolific author, followed by Gosseries O and Owen AM, two of whom are from the same group, the University of Liege, Belgium (Lauseys S and Gosseries O). The top three cited authors were Giacino JT, Laureys S, and Schnakers C, suggesting that they are the most representative researcher in the DoC field. It was evident from the network analysis that the top 10 most productive authors or cited authors were all from developed countries, Belgium appears to be a pivotal country in the field of DoC. 

The United States and the University of Liege were the most influential countries and institutions in the DoC field, with 534 and 303 articles, respectively. Of note, the University of Liege and University Hospital Liege, ranked first and second with the highest number of publications, respectively, in part because the literature for both institutions was signed by the same author. Significantly, the USA had the highest centrality, showing that it played a pivotal role in this field. European and American countries have made remarkable contributions. Through co-occurrence analysis, we found cross-country and cross-author collaborations; however, such collaborations were more commonly found in developed countries. The result was explainable because more medical support was available in developed countries, which encouraged researchers to explore this field. Although scholars from Asian countries are also increasingly emerging in DoC, their performance has not been prominent in centrality. 

In terms of journal distribution, the results show that Brain Injury is the most published journal on DoC research, followed by Frontiers in Neurology and Frontiers in Human Neuroscience. Furthermore, Neurology is the most co-cited journal, suggesting these journals are highly recommended in the DoC field. 

Through the analysis of country, institution, and author co-occurrence networks, we found strong collaboration among scholars from different countries and institutions. However, research progress on DoC was relatively slow in developing countries. Strengthening cooperation between countries can accelerate the development of the discipline and produce more high-quality publications. To promote the development of DoC disciplines, strengthening cooperation among countries, especially between developed and developing countries is still urgent to need.

### 4.2. Recent Hot Issues in DoC Research

Keywords co-occurrence and co-cited reference cluster analysis can identify hotspots in the research field [21]. DoC patients are mainly classified into VS and MCS according to different states/levels of consciousness. The top 10 most frequently occurring keywords contain words related to the state of consciousness (Table 4). Combining with the cluster analysis of literature co-citations results (Figure 4), we could conclude that current research in the DoC field focuses on distinguishing the level of consciousness, and early recognition of consciousness, especially in intensive care units (ICUs). We discussed the current hotspots of DoC from two perspectives: diagnosis of the level of consciousness in DoC patients and detection of early residual awareness of brain injury at the ICU bedside.

#### 4.2.1. Diagnosing the Level of Consciousness in DoC Patients 

Distinguishing the vegetative state from the minimally conscious state is a great challenge for clinicians. Currently, the clinical misdiagnosis rate is as high as 40% [6,22]. Diagnosing the state of consciousness accurately and recognizing the residual consciousness in the early stage of the severely brain-injured patient is of great significance [19], which could affect the clinician’s decision-making greatly. To reduce misdiagnosis DoC patients need serial standardized neurobehavioral assessments [18]. In 1991, Giacino et al., inspired by the poor performance of the Glasgow coma scale, proposed the Coma Recovery Scale (CRS) for the behavioral diagnosis of DoC patients [23]. Subsequently, they again proposed the revised version in 2004 to further improve CRS, namely CRS-R [24]. Wannez S and his colleagues confirmed that behavior CRS-R scores were influenced by fluctuations in clinical characteristics in patients with DoC. Therefore, it is recommended that patients with DoC undergo at least five evaluations within 10 days to reduce the impact of fluctuations in responsiveness [16]. Meanwhile, after clinical validation and comparison, CRS-R is strongly recommended as a standardized behavioral assessment for DoC patients [3,25].

In addition to the classification methods based on behavioral assessment indicators, neuroimaging and electrophysiology methods are also used as auxiliary measures for DoC diagnosis. More novel indicators were subsequently proposed [26], including the perturbational complexity index, bispectral index, and entropy, etc. Some other components of Event-Related Potentials were extracted from EEG, e.g., Mismatch negativity, and P300. TMS combined with EEG is an emerging assessment technique that allows researchers to measure local and global cortical activity directly [27]. The perturbational complexity index based on examination of the EEG response to TMS has been proven a good approach for identifying states of consciousness and unconsciousness [28], but more clinical studies were still needed to confirm its efficacy.

In neuroimaging aspect, fMRI can dynamically map changes in brain activity by detecting the blood-oxygen-level-dependent signal. Several studies demonstrated either resting state fMRI or Event-Related-fMRI successfully differentiating the levels of consciousness. Resting-state fMRI detection of the default mode network (DMN) revealed that altered functional connectivity positively correlated with the extent of consciousness [15,29]. In addition, positron emission tomography imaging has been proven to have excellent sensitivity in distinguishing MCS from VS [14].

All of these indicators and methods are considered prospective in differentiating the level of consciousness. Nevertheless, since each technique has certain advantages and disadvantages, multimodal assessment is strongly recommended to reduce the rate of misdiagnosis [25].

#### 4.2.2. Detecting Residual Awareness after Early Brain Injury at the ICU Bedside

Early monitoring of patients in the ICU and the management of critical complications have a favorable impact on the prevention and treatment of impaired consciousness [30]. These complications such as seizures, pulmonary infections, and cardiac arrests may occur during ICU, leading to coma or exacerbation. Also, detecting DoC patients’ residual awareness is vital in the early stage of brain injury, especially in ICU. Due to the high rate of misdiagnosis, the true levels of consciousness of those patients with lower levels of consciousness are often underestimated [13]. They could follow commands through brain activity, rather than through words or movement [31], which was inconsistent with the behavioral assessment. According to the report, about 15% of patients with low levels of consciousness may have covert consciousness [32]. 

Fernández-Espejo et al. believed a dysfunction in thalamocortical circuits is the neural explanation for the loss of external responsiveness in some severely brain-injured patients [33]. Schiff ND suggested the term cognitive motor dissociation (CMD), also known as a functional locked-in syndrome [34], for patients with unrecognized awareness at the bedside, but it may be detected by neuroimaging or electrophysiological techniques [35,36]. However, finding more evidence to explain possible underlying mechanisms of CMD was still challenging. Detecting covert consciousness was also discussed in another article [30], in which the importance of early identification of covert awareness in the ICU was emphasized. Early identification of such patients facilitates further disease prognosis [17]. Patients with CMD detected early in brain injury had a better prognosis at 12 months [32]. Notably, Egbebike J et al. suggested CMD could serve as a sign of recovery, and a biomarker of the residual integration function in the injured brain [37]. 

Two methods are frequently used to detect covert awareness: event-related-fMRI or event-related -EEG to capture brain activity. Patients were told to imagine playing tennis or other tasks and trying to follow the command during fMRI, which has been proven as an effective tool in detecting CMD patients [31]. Another method is the EEG-based brain-computer interface [38]. Yet, the accuracy of the brain-computer interface in identifying CMD patients was encouraging. In short, optimizing the experimental paradigm and identifying the residual consciousness of DoC patients are still major access points for current research. 

### 4.3. Emerging Trends of DoC Research

Through our review of research topics for DoC in the recent 10 years, based the Figure 5B and Figure 6, burst keywords can indicate emerging trends and timeline analysis can visually display the changing trends of the research topic over time. Combining recently emerged burst keywords and spotlighted clusters, we concluded two emerging trends in the DoC field, including exploring the mechanism of consciousness recovery and neuromodulation interventions for DoC patients.

#### 4.3.1. Exploring the Mechanism of Consciousness Recovery

Consciousness is the experience of one’s environment and inner self. There are four prominent consciousness theories: higher-order theories, global workspace theories, re-entry and predictive processing theories, and integrated information theory [39]. The neurobiological mechanism of consciousness formation is one of the most important scientific questions. The mechanism of consciousness recovery remains unclear. Electrophysiological and neuroimaging-based studies suggested that consciousness recovery depends on thalamocortical, corticocortical, and thalamostriatal nuclear connections [4]. Notably, the mesocircuit model, which is focused on central thalamus and frontostriatal interactions, emphasized the great importance of the frontoparietal network in restoring consciousness, providing a possible perspective to explore the recovery mechanism of DoC [40]. Interestingly, some patients underwent bilateral frontal lobectomy and remained well-conscious. Furthermore, there was a patient with deficits in cognitive function supported by the frontal lobes after extensive frontal lobe damage, but her consciousness and perception were preserved [41]. However, according to recent research, the posterior cerebral cortex (temporal, parietal, and occipital) may directly contribute to defining the nature of consciousness [42]. 

Bodien et al. attempted to explain the mechanism of consciousness recovery in terms of brain functional network connectivity [43]. They found that brain networks were associated tightly with consciousness, especially DMN, of which integrity represents a fundamental feature of brain function [44]. Activation of DMN was absent in brain-dead patients and decreased significantly in VS patients, while slightly decreased in MCS patients compared to healthy patients [45], indicating the importance of the DMN for consciousness. Similarly, a few studies have reported an increase in brain metabolic activity and functional network connectivity when patients move from a low to a high level of consciousness. Chennu S and Perri CD explored possible mechanisms from the perspective of brain metabolism in patients with DoC. They found that brain networks based on EEG or MRI showed strong correlations with brain metabolism [46,47]. 

#### 4.3.2. Neuromodulation Interventions for DoC Patients 

The level of consciousness classification and early identification of patients with DoC are all to accurately judge the patient’s condition, and these are closely related to the patient’s prognosis and the choice of treatment methods. Neuromodulation has become a hot spot in treating patients with DoC. Invasive methods refer to deep brain stimulation (DBS), Vagus nerve stimulation, spinal cord stimulation, etc. Non-invasive methods mainly include repetitive transcranial magnetic stimulation (rTMS) and transcranial direct-current stimulation (tDCS) etc. 

DBS has been approved to restore certain cognitive and motor functions by stimulating deep brain thalamic nuclei. It was widely used in neuropsychiatric disorders and neurodegenerative diseases, e.g., depression, and Parkinson’s disease. Some studies reported that direct or indirect damage to the thalamus seems to be a major cause of DoC after brain injury [48], and some specific deep brain nuclei in the thalamus are associated with arousal, which is proposed as significant intervention targets for DoC patients [49]. It is now believed that the delivery of electrical impulses via DBS to circuits in the anterior forebrain can encourage synaptic activity in its related structures, thereby altering the arousal regulation system and making recovery of cognitively mediated behavior easier [50]. The midbrain circuit model provides a possible mechanism for DBS to treat DoC, and the midbrain thalamus is often an interesting target concerning the brainstem and frontal lobes, which are central to arousal and attention [51]. Redinbaugh et al. found that stimulating the central lateral thalamus with DBS can wake monkeys under anesthesia, thus raising the level of consciousness [52]. The efficacy of DBS had also been demonstrated in DoC patients, Schiff et al. verified DBS could improve behavior-responsive function and awareness in MCS patients after severe brain-injured [53]. 

Furthermore, rTMS and tDCS could regulate cortical excitability, restoring consciousness [54,55]. Finding effective and individualized targets is a challenge for using non-invasive neuromodulation techniques in DoC patients. The left dorsolateral prefrontal lobe and primary motor cortex were the most studied rTMS targets in DoC patients [56,57]. However, there is no unified view of its clinical efficacy. Some randomized controlled trials seem to suggest that tDCS performed better in recovering cortical activity and functional connectivity in MCS than VS [55,58], and the target over the left dorsolateral prefrontal lobe was more effective than other targets. Nevertheless, the mechanism by which neuromodulation techniques are used to restore consciousness remains unclear and still needs further research. In general, non-invasive neural regulation can restore consciousness to some extent and is a promising treatment for DoC. Meanwhile, exploring effective, individualized parameters and targets is the direction for future research in DoC. 

## 5. Strengths and Limitations

As far as we know, we are the first ones to offer a bibliometric analysis that unveils the intellectual framework of nearly a decade of research on DoC. We comprehensively explored the current research status in DoC from a longer time dimension and tried to mine future research directions from the results of the quantitative analysis. The above analysis can provide researchers with a broad vision of DoC studies. Yet, there are still some limitations to be noted. Due to various limitations in the literature selection process, some publications were not included in the study. We only collected English publications from SCI-EXPANDED of WoS. Thus, the results we displayed in this study may not be comprehensive. Future studies need to incorporate more databases with fewer restrictions. Therefore, future updates on the study are necessary. More document types and language formats should be considered to reduce bias. In addition, the period analyzed in this study is relatively short, from 2012 to 2021, leading to incomplete results. Despite these limitations, we still believe that this study can shed light on the research trends and hot issues in the field of disorders of consciousness. 

## 6. Conclusions

This study uses bibliometric tools, with CiteSpace and Gephi software as visualization tools, though quantitatively analyzing DoC research from 2012 to 2021, reveals the knowledge structure of research on disorders of consciousness in the past 10 years, summarizes the development status of DoC, mines the possible directions for future research from the results, which provides researchers with a new perspective. Combined with the above analysis, we can conclude that diagnosing accurate levels of consciousness and identifying early residual consciousness are current topic issues in the field of DoC. The emerging trends focus on exploring the neural mechanism of consciousness recovery and seeking effective treatment for DoC patients, especially neuromodulation methods. In general, our findings are based on the perspective of the country, institution, author, keyword co-occurrence analysis, and references co-citation analysis. Through this article, researchers can gain a more comprehensive understanding of this field. Meanwhile, it could provide a reference for future research.

## Figures and Tables

**Table 1:** The most active author and co-cited author in DoC field.

Rank	Count	Author	H-Index	Country	Rank	Count	Co-Author	H-Index	Country
1	163	Laureys S	96	Belgium	1	1026	Giacino JT	50	USA
2	62	Gosseries O	41	Belgium	2	804	Laureys S	96	Belgium
3	56	Owen AM	85	Canada	3	568	Schnakers C	96	USA
4	53	Chatelle C	29	Belgium	4	482	Monti MM	22	USA
5	52	Thibaut A	26	Belgium	5	417	Schiff ND	44	USA
6	43	Giacino JT	50	USA	6	405	Ashwal S	61	USA
7	37	Bramanti P	52	Italy	7	400	Boly M	65	USA
8	37	Calabro RS	28	Italy	8	399	Owen AM	85	Canada
9	35	Naro A	23	Italy	9	343	Bruno MA	44	Belgium
10	33	Schnakers C	96	USA	10	337	Jennett B	48	UK

**Table 2:** Top 10 of the most influential journals of DoC research.

Rank	Journal	Impact Factor (2021)	JCR (2021)	Count	Co-Journal	Impact Factor (2021)	JCR (2021)	Count
1	Brain Injury	2.167	Q2	101	Neurology	11.8	Q1	1215
2	Frontiers in Neurology	4.086	Q2	53	New England Journal of Medicine	176.079	Q1	930
3	Frontiers in Human Neuroscience	3.473	Q2	44	Archives of Physical Medicine and Rehabilitation	4.06	Q1	911
4	PloS One	3.752	Q2	39	Lancet	202.731	Q1	879
5	Archives of Physical Medicine and Rehabilitation	4.06	Q1	37	Brain Injury	2.167	Q2	832
6	Neuroimage-Clinical	4.891	Q2	37	Brain	15.255	Q1	819
7	Brain Sciences	3.333	Q3	36	Neuroimage	7.4	Q1	714
8	Clinical Neurophysiology	4.861	Q2	33	Ann Neurol	11.274	Q1	623
9	Journal of Neurotrauma	4.869	Q2	33	J Neurol Neurosurg Ps	13.654	Q1	615
10	Frontiers in Neuroscience	5.152	Q2	29	Clin Neurophysiology	4.861	Q2	614

**Table 3:** The top 10 reference co-citation in terms of frequency.

Rank	Co-Cited References	Impact Factor (2021)	Count
1	Monti MM, 2010, NEW ENGL J MED, V362, P579, DOI 10.1056/NEJMoa0905370 [11]	176.079	178
2	Laureys S, 2010, BMC MED, V8, P0, DOI 10.1186/1741-7015-8-68 [12]	11.15	139
3	Schnakers C, 2009, BMC NEUROL, V9, P0, DOI 10.1186/1471-2377-9-35 [6]	2.903	126
4	Giacino JT, 2014, NAT REV NEUROL, V10, P99,DOI10.1038/nrneurol.2013.279 [3]	0.29	124
5	Cruse D, 2011, LANCET, V378, P2088, DOI 10.1016/S0140-6736(11)61224-5 [13]	202.731	116
6	Stender J, 2014, LANCET, V384, P514, DOI 10.1016/S0140-6736(14)60042-8 [14]	202.731	98
7	Vanhaudenhuyse A, 2010, BRAIN, V133, P161, DOI 10.1093/brain/awp313 [15]	15.255	92
8	Wannez S, 2017, ANN NEUROL, V81, P883, DOI 10.1002/ana.24962 [16]	11.274	89
9	Bruno MA, 2011, J NEUROL, V258, P1373, DOI 10.1007/s00415-011-6114-x [17]	6.682	87
10	Giacino JT, 2018, NEUROLOGY, V91, P450, DOI 10.1212/WNL.0000000000005926 [18]	11.8	87

**Table 4:** The top10 keywords ranked by frequency and centrality.

Rank	Count	Keyword	Centrality	Keyword
1	784	vegetative state	0.56	awareness
2	399	minimally conscious state	0.38	connectivity
3	369	disorder	0.37	own name
4	318	disorders of consciousness	0.29	vegetative state
5	307	recovery	0.28	electrical stimulation
6	255	traumatic brain injury	0.27	prognostic value
7	235	brain injury	0.26	recovery
8	233	coma	0.26	complexity
9	165	persistent vegetative state	0.23	transcranial magnetic stimulation
10	147	awareness	0.23	modulation

## Data Availability

All original data on this article will be provided without reservation, and details can be obtained from the corresponding author.

## Supplementary Materials

The following supporting information can be downloaded at: https://www.mdpi.com/article/10.3390/medicina59010078/s1. Supplementary Materials Spreadsheet S1: Basic information of the data.
